# Fatherhood and Elite Athletics: Sacrifice, Selfishness, and Gaining “Dad Strength”

**DOI:** 10.1177/10608265231204564

**Published:** 2023-11-20

**Authors:** Sydney V. M. Smith, Francine E. Darroch, Audrey R. Giles, Dylan Wykes

**Affiliations:** 1 School of Human Kinetics, Faculty of Health Sciences, 70363 University of Ottawa, Ottawa, ON, Canada; 2 Department of Health Sciences, Faculty of Science, 120862 Carleton University, Ottawa, ON, Canada; 3 Mile2Marathon Coaching Inc., Ottawa, ON, Canada

**Keywords:** involved fatherhood, elite athlete, caring masculinities, parents, athletics

## Abstract

This study contributes to a growing body of scholarly discussions around the many aspects and challenges of combining parenthood with elite-level sport, with a particular focus on the experiences of male elite athletes who are fathers. We used a caring masculinities theoretical framework, community-based participatory research, and semi-structured interviews to explore the experiences of 10 elite/international and world-class athletes (*n* = 9 fathers, *n* = 1 expectant father). Through reflexive thematic analysis, we identified three main themes: fatherhood can (1) improve and (2) impede elite athlete-fathers’ athletic performance; and (3) athlete-fathers experience a trade-off between athletic performance and fatherhood responsibilities. Our findings underscore the ways in which male athletes’ experiences with parenthood reflect the new era of involved fatherhood and are analogous to some of the identity tensions that have been reported with regard to the experiences of elite female athletes who are pregnant and/or mothers. Recognizing the impact of children on male athletes’ athletic careers and the parallels between fatherhood, motherhood, and elite sport may lead to better support for athlete-fathers while also contributing to diminishing the expectation that women are primary caregivers to children.

Elite athletes are required to constantly manage various expectations from athletic performance to nutrition, sleep, and recovery, which can trickle into “life-consuming territory” (Boniface, 2017, para. 3). Though a career in professional sport leaves little room for other major life events – such as parenthood – this is not to say that this type of balance is an impossible feat. Indeed, numerous scholars and athletes have recently explored and publicly expressed several elite athlete-parents’ successful journeys as they have pursued parenthood amid a career in elite-level sport (Darroch et al., 2022a; Davenport et al., 2022; Massey & Whitehead, 2022; Tekavc et al., 2020). Such actions have brought many concerns and issues to light including the severe lack of support and resources that are (un)available within the sports industry for pregnant/parenting elite athletes – particularly athlete-mothers (Darroch et al., 2016, 2019; Felix, 2019; Montaño, 2019; Scott et al., 2022; Smith et al., 2023).

There is a scarcity of attention concerning the experiences of male elite athletes who are fathers. Notably, this gap in understanding of athlete-fathers’ experiences coincides with recent shifts in men’s roles in childcare (Machin, 2015). Such shifts have been exemplified by recent increases in the amount of time that fathers spend engaging in caregiving duties as well as in the development of emotional relationships with their children (Wall & Arnold, 2007). As highlighted by Scambor et al. (2014), “[m]en’s attitudes have slowly shifted from clear breadwinner roles toward care-integrating models (especially fathering) over the past few decades” (p. 560). With this in mind, there is a need for further research into the ways in which male athletes in particular are affected by fatherhood and dominant societal narratives that portray fathers as secondary parents (Stevens, 2015; Sunderland, 2000; Wall & Arnold, 2007). The purpose of this study was therefore to explore male elite/international and world-class^1^ athletes’ accounts of their fatherhood experiences and the impact of children on their high-performance athletic careers.

Below, we present a review of the relevant areas of literature, followed by descriptions of our use of a caring masculinities theoretical framework, community-based participatory research (CBPR) methodology, semi-structured interviews, and reflexive thematic analysis (RTA). We then describe our results of this study (which are encompassed within three main themes: fatherhood can (1) improve and (2) impede elite athlete-fathers’ athletic performance; and (3) athlete-fathers experience a trade-off between athletic performance and fatherhood responsibilities), followed by a discussion of our findings as well as our conclusions.

## Literature Review

### Men’s Roles in Parenting

There has been a substantial shift over the years in societal perceptions of fatherhood (Meleagrou-Hitchens & Willig, 2022) as well as gender roles in parenthood (Oláh et al., 2021). Particularly in Western societies, the longstanding “traditional” role of a father has been denoted by an idealized ability to protect and provide for one’s children and family (Buchler et al., 2017), while the “new” father insinuates an expectation for men to be more nurturing than in the past and to care for their children both physically and emotionally (Tichenor et al., 2011; Wall & Arnold, 2007). A major caveat within the shifting of parenting roles is that fathers are discursively produced as falling into one of two classifications: the traditional father versus the new father. Reducing fatherhood to two mere classifications can be problematic given that men tend to fluctuate between these two classifications at different points in time (Lewington et al., 2021). Scholars have thus recently advocated for fatherhood involvement to be considered along a continuum (Bataille & Hyland, 2023).

### Impacts of (Involved) Fatherhood on Men

The many ways in which fatherhood impresses upon men – including varying forms of emotional changes (Chin et al., 2011) and sleep disturbance (Wynter et al., 2020) – are often overlooked given the significant focus that is placed on mothers’ experiences. Failing to acknowledge how fatherhood may impact men can perpetuate the assumption that parenting responsibilities are reserved for women only, in addition to potentially leaving fathers feeling ill-prepared for the transition into parenthood (Condon et al., 2004) and with a lack of resources geared towards them (Baldwin et al., 2018; Darwin et al., 2017).

An element of particular concern is the level of stress that fathers experience, which can be exacerbated by modified sleep patterns that are characteristic of new parenthood (i.e., a lack of sleep as well as poor quality and/or quantity of sleep). The combination of these factors can have severe impacts on fathers’ mental health and wellbeing (Da Costa et al., 2019; Philpott et al., 2019). It is equally as important to note that a significant number of men also experience many positive changes through their transition into fatherhood, including newfound feelings of confidence (Kotila & Kamp Dush, 2013) and motivation (Keizer et al., 2010), as well as improved emotional, mental, and even physical health (Astone & Peters, 2014; Knoester & Eggebeen, 2006; Knoester et al., 2007; Kotila & Kamp Dush, 2013). However, a potential barrier to realizing the benefits of involved fatherhood are the challenges that can arise when balancing family with career.

Fathers’ pursuits towards work-family balance are often hindered by conflicting expectations, which can leave fathers in a position where they might experience feelings of guilt (Gonçalves et al., 2017) for decreasing work hours to spend time with children (Moran & Koslowski, 2019) or for sacrificing time with their children to attend to work-related matters (Machin, 2015). Indeed, the various difficulties in establishing work-family balance are furthered within a work environment that is as intense, demanding, and emotional as high-performance sport (Taylor et al., 2019).

### Elite Athletics and Parenthood

A career as an elite athlete represents a work environment with which it can be difficult to balance other life elements, including family. Elite athletes who are pregnant/parenting are subject to a two-pronged “all or nothing” mentality wherein one side is driven by societal expectations that are rooted in discourses of parenthood, while the other side is primarily influenced by the sports industry. The scholars (e.g., Jackson et al., 2022; Palmer & Leberman, 2009; Smith et al., 2023) who have recently explored the many aspects of motherhood and elite athletics have collectively described the various pressures that many female athletes experience when navigating both motherhood and athletic obligations (Darroch & Hillsburg, 2017).

First, there is a form of pressure that stems from discourses of what it means to be a “good mother” in that good mothers “should invest swaths of time, money, energy, and emotional labor in intensively raising children” (Elliott et al., 2015, p. 352). Entrenched in an ethic of care (Miller & Brown, 2005), the good mother has also been described as one who makes sacrifices for her children (Florczak, 2004), which could include elite athletic commitments (Darroch & Hillsburg, 2017). Tied with the social construct of a good mother, elite athlete-mothers are simultaneously subjected to the problematic discourse that an athlete’s serious commitment to their athletic career must be illustrated by organizing everything around (and possibly sacrificing many things for) their sport (Spowart, 2021).

In considering gender equity matters in relation to parenthood, there is value in recognizing the ways in which understandings around mother-athletes in good motherhood are parallel to those related to the “good father” discourse. The good father ideal reflects the expectations that are often weaved in with those related to the non-traditional “new” or “involved” father, which prescribe men to be nurturing and to care for their children both physically and emotionally (Tichenor et al., 2011; Wall & Arnold, 2007). These expectations are in line with those of the good mother-athlete as an elite athlete’s pursuit to be a good father can come at the cost of being questioned for their genuine commitment to their athletic career (McGannon et al., 2018).

Despite vast advancements in recent work around motherhood and elite sport, there has yet to be an analogous scope of research aimed at recognizing how an elite athlete-father’s running career may also be affected by parenthood. The limited representation of the experiences of professional male athletes as fathers may be seen as a reflection of the view that women are primary caregivers (Locke & Yarwood, 2016; McGannon et al., 2018). Existing research that has focused on fathers in sport has included fathers’ influence on youth participation in sport (Coakley, 2006; McGannon et al., 2018; Pot & Keizer, 2016), how fathers construct and enact the meaning of a “good father” within the context of sport and leisure (Park & Kwon, 2019), family influences on sport participation (Dionigi et al., 2012), or has been in relation to either fathers as high-performance sport coaches (Graham & Dixon, 2014, 2017), or as athletic trainers (Rynkiewicz et al., 2021).

There are, however, a few key studies that have merged the realms of involved parenthood, family life, gender roles, and sport. Through an examination of fathers who compete in iron-distance triathlons, Cohen (2016) highlighted the “iron dad” identity as one that requires a “juggling act” involving numerous social and psychological challenges as they negotiate sport, work, and family life. Andreasson et al. (2018) also considered the experiences of amateur Ironman triathletes (specifically 14 men and 12 women) and found that when it came to childcare and family roles, nearly all participants described themselves as being equal with their partner/spouse in theory but reported that the pursuit of such equality is often difficult in practice.

McGannon et al.’s (2018) study has been influential in exploring fatherhood in relation to elite professional sport. These authors explored the media representations of one male athlete’s journey as both a father and high-profile athlete and identified two major identities, both of which were shaped by one central narrative of involved fatherhood: the “good father” and the “new and improved athlete father” (p. 676). The authors underscored how these major identities constructed fatherhood in relation to sport in ways that both affirmed but also resisted cultural ideals concerning gender, sport, and parenthood. McGannon and colleagues also identified the portrayal of work-family tensions between being a good father versus a committed athlete – a finding that is akin to tensions described in relation to elite female athletes and motherhood (Darroch & Hillsburg, 2017; McGannon et al., 2015). Nevertheless, it is important to note that McGannon et al.’s (2018) findings were produced from news media constructions, rather than an athlete’s stated lived experiences. Tekavc et al.’s (2015) study, in which three of their 12 participants were men who became fathers during their professional athletic careers, provided a glimpse into athlete-fathers’ experiences of added fatigue and feelings of guilt, as well as how having children can represent an “additional source of motivation” (p. 36) towards their sport performances.

Though these scholarly works represent significant additions to the literature, especially in light of developing cultural meanings of “the new fatherhood,” there is still a gap with regard to further understanding the complexities of fatherhood, elite professional sport, and gender equity. Our main objective was thus to contribute to attending to this body of knowledge by exploring how male world-class and elite/international-class athletes in the sport of athletics experience fatherhood and the impact of children on their athletic careers. To our knowledge, this is the first CBPR study to look at the experiences of multiple fathers who are such high calibre professional athletes.

## Theoretical Framework

We employed a theoretical framework informed by Elliott’s (2016) concept of caring masculinities to explore elite male athletes’ constructions of their fatherhood experiences. Elliott conceptualized caring masculinities as “masculine identities that reject domination and its associated traits and embrace values of care such as positive emotion, interdependence, and relationality” (p. 240). It is imperative to note that gender relations are driven by human social practice, which differ and fluctuate across social settings and throughout time. Hence, masculinity is also varied and constantly changing, giving way to multiple social constructions. In turn, it is not one single *masculinity* that is encompassed within this framework but rather multiple *masculinities*. This notion is fundamental in recognizing that there is “variation among men and expressions of masculinities” (Elliott, 2016, p. 245).

Elliott’s (2016) caring masculinities framework carries important implications for gender equality by way of a nuanced lens for examining traditional gender roles and practices (Lee & Lee, 2018). Connell's (1987, 1995; Connell & Messerschmidt, 2005) concept of hegemonic masculinity, which has played a major role in scholarly discussions around men, gender, and social hierarchy (Connell & Messerschmidt, 2005), was influential in shaping Elliott’s (2016) theorization of caring masculinities. Connell (1995) defined hegemonic masculinity as the “configuration of gender practice which embodies the currently accepted answer to the problem of the legitimacy of patriarchy, which guarantees (or is taken to guarantee) the dominant position of men and the subordination of women” (p. 77). Elliott (2016), however, stressed that the concept of hegemonic masculinity bears harmful costs through the articulation of unattainable ideals to which men are seemingly expected to adhere, which creates a barrier to men’s participation in gender equality. A key tenet of the caring masculinities framework is thus the rejection of domination; indeed, as asserted by Elliott (2016), to “ensure the absence of domination” is to “ensure the presence of equality” (p. 252). Hence, our employment of a theoretical framework informed by caring masculinities allowed us to consider the ways in which elite male athletes’ constructions of their fatherhood experiences contrast traditional masculinities and consequently contribute to changes in socially constructed meanings of gender (Elliott, 2016) within elite athletics.

## Methodology

We used principles of CBPR to inform our approach to this study, which, to our knowledge, is the first of its kind in this area of research. Rooted in a participatory research framework, CBPR is suited for exploring research objectives that are geared towards supporting men, women, or groups who may be ignored (Reid, 2004). This methodology therefore allowed us to take into consideration how elite male athletes are almost never recognized as athlete-fathers, even if they have children (de Haan & Knoppers, 2020) and, in turn, the direct impact that this can have on male athletes who are fathers. Recognition of elite athletes as fathers may be significant in contributing to alleviating the burden of parenting responsibilities that is placed on athlete-mothers. Importantly, CBPR is centred on the collaboration between two main parties: a research team and members of a community which the study is focused on (Roberts, 2013).

CBPR researchers must also recognize their own positionalities in relation to their research (Muhammad et al., 2015). The first author is a national-level track athlete; author two is a mother, a former elite-level runner who competed on international teams as an under-20 athlete, and she is married to a 20XX [anonymized] Olympic long-distance runner; author three was a national-level varsity runner; and author four is an advisory board member, a father, and an Olympian. Our intimate familiarity with the sport of athletics is also further extended within academia specifically, which is evidenced by our previous contributions to literature on parenthood (particularly motherhood) and eliteathletics (e.g., Darroch & Hillsburg, 2017; Darroch et al., 2016, 2019, 2022a, 2022b; Giles et al., 2016; Scott et al., 2022; Smith et al., 2023). Our nuanced understandings of athletics as participants, researchers, and fans have hence played a key role in conducting this study.

Moreover, in accordance with the central collaborative tenet of CBPR, this study involved working closely with a community advisory board (CAB). Conducting CBPR in partnership with a CAB empowers community members and leads to opportunities for research that can directly benefit the study community (Roberts, 2013). Our CAB was made up of five elite/international and world-class distance runners who are all parents (two fathers and three mothers), who each contributed to our research process and provided guidance based on their lived experiences as elite athlete-parents.

## Methods

This study received approval from both the [Anonymized] as well as the [Anonymized] Research Ethics Boards. With guidance and help from our CAB, we drew a purposive sample of athlete-participants, complemented by snowball sampling techniques (Bowling, 2014; DeCarlo, 2018) to recruit participants who met the study inclusion criteria: understand and speak English, current or expectant father, and currently (or within the last five years) of elite/international to world-class caliber in any middle- or long-distance running event according to the classification framework defined by McKay et al. (2022). We recruited 10 participants for this study: all hail from three high-income countries, all have a partner/spouse of the opposite sex, and all but one are white. In accordance with McKay et al.’s (2022) criteria, six of the participants were Tier 4 (Elite/International Level) and four were Tier 5 (World Class). The 10 participants have, combined, competed at 14 Olympic Games and 15 World Championships. We assigned all participants a pseudonym (see Table 1) to protect their identity and omitted other identifying details where necessary.

**Table 1.** Participant Characteristics (*N* = 10)

Category	Pseudonym	Classification
Father	Colin	Elite/International level
Father	Zach	Elite/International level
Father	Phil	Elite/International level
Father	Jonathan	Elite/International level
Father	Brian	Elite/International level
Father	Dennis	World class
Father	Jake	World class
Father	Remy	World class
Father	Cameron	World class
Expectant father	Eric	Elite/International level

The first and second authors conducted semi-structured interviews to gather data. Our use of this method allowed us to ask specific questions while also making room for each athlete to share any other thoughts (Blee & Taylor, 2002) concerning the questions we posed. We asked participants specific questions related to their own experiences of navigating a high-performance athletic career with fatherhood and what this balance might look like for them with family responsibilities, training camps, competitions, and day-to-day training. Importantly, these questions were designed to reflect key nuances that were underscored by our CAB members and their lived experiences navigating their own elite athletic careers in tandem with parenthood. We conducted the interviews virtually through Zoom, an online video platform, between April 2021 and January 2022. The interviews lasted from 45 to 95 minutes. To thank participants for their time, each individual received a $25 gift card. Each interview was audio recorded and transcribed verbatim. We sent each participant their transcript and allowed them the opportunity to omit or clarify any text, if they deemed this necessary. One participant requested that his transcript not be sent to him as he did not feel that he would have any revisions to make, and none of the remaining nine participants asked for any changes to their transcripts.

## Analysis

We analyzed our data using RTA as this approach aligned well with our use of a caring masculinities theoretical framework and CBPR methodology. Building on the foundations of Braun and Clarke’s (2006) initial articulation of thematic analysis, RTA allows for researchers to identify, analyze, and interpret patterns of meaning (i.e., themes) within qualitative data and is distinguished from several other approaches to analysis on the basis of its flexibility (Braun & Clarke, 2019; Clarke & Braun, 2017). Braun and Clarke’s (2006) six-phase approach to thematic analysis both “celebrate[s] the flexibility” (Braun & Clarke, 2006, p. 78) of thematic analysis but also provides a corresponding degree of guidance to be able to appropriately perform analysis in a manner that fits with specific research objectives and data (Braun & Clarke, 2006; Patton, 1990). We followed Braun and Clarke’s (2019) reflexive approach to thematic analysis because it places a distinct focus on researchers’ roles in knowledge production. RTA can be used to reflect researchers’ interpretations of a dataset, all the while factoring in the underlying theoretical assumptions that inform the study in addition to the perspectives and positionings that the researchers themselves bring to the analysis process (Braun & Clarke, 2019). Our use of RTA was thus a key contributing factor to the novelty of our work in this area of research given our CBPR approach and the close involvement of our CAB throughout each stage of our research process.

Our analysis process was a collaborative effort between all four authors and the CAB members, which included familiarizing ourselves with the data; generating codes; and developing, reviewing, and defining themes. We used the qualitative software package NVivo 12^TM^ to assist our data management and organization throughout our analysis. The first author reviewed, transcribed, and coded each interview transcript prior to the initial theme development and review stages, which were carried out by authors one, two, and three. Some examples of the codes identified included “fatherhood as motivation,” “fatherhood as distraction,” “balancing responsibilities,” “sleep,” “sacrifice,” “traveling,” and “guilt.” In collaboration with the fourth author in addition to guidance, debriefs, and multiple reviews from our CAB, we defined and named key themes, which, importantly, was a part of a recursive (as opposed to a linear) process throughout each of these phases of analysis (Braun & Clarke, 2006, 2019). The final phase of our analysis is represented by this paper as we share our results within an “analytic narrative” that “illustrates the story” that we are telling about our data (Braun & Clarke, 2006, p. 93). In short, our analysis led to the collaborative construction of three main themes, which we describe below.

## Results

We identified three main themes: (1) fatherhood improves elite athlete-fathers’ athletic performance; (2) fatherhood impedes elite athlete-fathers’ athletic performance; and (3) elite athlete-fathers experience a trade-off between athletic performance and fatherhood responsibilities. The juxtaposition of the first two themes sheds light on the importance of acknowledging the tensions that exist between them, which are further highlighted within the third theme and, in turn, contribute to understanding the varying experiences of male elite runners as they navigate fatherhood.

### Fatherhood Improves Elite Athlete-Fathers’ Athletic Performance

All participants asserted that becoming a father was a life-changing experience, which had positively contributed to athletic performance. Remy described his introduction to fatherhood as “the best thing that has ever happened to me, that’s for sure.” He welcomed the new responsibilities that came with parenthood: “Athletics is a very small bubble … it’s nice to escape it once in a while and having a child definitely makes you do that.” Brian also explained that he had a newfound understanding of what it meant to gain “dad strength”: “I think having a kid definitely gives you something else to work for beyond yourself … and something to maybe think about when things get hard in a race or a workout or whatnot.”

Dennis echoed these sentiments when he described a shift in his approach to workouts after becoming a father:

Every run … was much more important … I had to maximize my time. … It was like, okay, I’m missing whatever amount of time away from [my daughter], this has to be important. And from [the year my daughter was born] on, I think were the best four or five years in my career.

Similarly, Jake noted that having to navigate new challenges is worth the significant rewards brought on by fatherhood: “I’m struggling somewhat … but for me, [fatherhood is] worth every second of it … it gives you a drive and purpose and perspective on life that you wouldn’t have without a child.” Jake also added that having his daughter in attendance for competitions would be an “added motivational boost” to his athletic performance. Zach expressed an analogous position: “When my family is there, I feel like it’s less stress … I think having your family there … it takes that sort of edge off … I think it could propel me spiritually.”

Phil shared how improvements to athletic performance that stem from fatherhood may be rooted in his position as not just a father but also as a role model:

I do think that there’s something valuable in [my son watching me train]. Like motivation-wise for me. … Like hopefully those things start to register and like what goes into what we do [as elite athletes] and that would be, I think, pretty powerful.

Fatherhood thus played a very important role in stimulating significant feelings of motivation towards training, competing, and overall athletic performance for the participants.

### Fatherhood Impedes Elite Athlete-Fathers’ Athletic Performance

Several of the fathers underscored some of the major adjustments that they endured upon becoming fathers, many of which provoked impediments to performance. Some of the fathers especially expressed concern with regard to substantial disruption to their sleeping patterns. Zach stressed that, “before kids … getting a bad night’s sleep might happen once a month. And then with kids … it can happen five nights in a week.” Jonathan described how these poor sleeping patterns can accumulate: “the moment you get into a deficit, the moment you have one bad night’s sleep … you’re playing catch up and you never get it back.” The overall exhaustion that many of the fathers faced impeded their athletic performance in various ways. Jake noted:

[The] first four weeks of becoming a dad was bloody hard. … It was sort of surviving on … six hours [of sleep] on a good day … it was really, really difficult. … And I think I tore my calf through lack of sleep … and then I was expecting to sort of train twice a day … I was just so beat up and run down. … I was just mentally, just really drained.

Cameron echoed a similar sense of exhaustion when he shared that his son was born “right in the middle of track season.” He elaborated on the challenges that came with navigating this experience:

It was exhausting. And, honestly, it probably kept me from making the World Championship team [that year]. … [My wife and I] got home from the hospital [after our son was born] at like midday, and I got a flight to LA that next morning to race the day after. And my body was just shocked … that summer [season]. I was just toast the whole time.

Several of the participants also explained how daily parenting responsibilities can hinder athletic performance in small yet consistent ways. For example, when discussing efforts to fit in training as well as other athletic-related routines (e.g., stretching or recovery practices) amid a full day of parenting duties, Zach claimed that “things are just a bit more rushed” or “I just don’t do [the exercises].” Indeed, having to repeatedly neglect small parts of training sessions can, over time, have severe effects on overall performance. Jonathan also outlined the lifestyle adjustments that are required to be able to adapt to the daily duties of parenting, which involve having to “let go of some of our old habits,” including “routine” and “set time[s]” for eating, sleeping, and recovery. Colin also discussed these issues in the context of travel:

Prior to being a parent, if I decided I wanted to go to a training camp … I could just go. I [could] literally just pick up, pack my car or buy a plane ticket, and go. I didn’t really have any responsibilities to anyone else.

For many of the fathers in this study, there were thus varying means through which fatherhood could impede athletic performance.

### Elite Athlete-Fathers Experience a Trade-Off Between Athletic Performance and Fatherhood Responsibilities

In balancing athletic commitments with fatherhood responsibilities, the participants emphasized that improving their performance in one of these areas often meant having to diminish their level of involvement in the other. While this trade-off was felt by all the fathers in this study, each of them expressed a slightly different approach to having to “divide up your energy” between fatherhood and athletic performance.

Many of the fathers emphasized “selfishness” as a key element in decisions around the trade-off between fatherhood and athletic performance. Dennis acknowledged, “We’re in a sport where track is very selfish, right? It’s you against everyone else. You have to take you first. … When you’re in season … everything is about you.” Many of the fathers described the act of making selfish decisions as the ticket to reaching full athletic potential – but that this comes at the cost of missing out on fatherhood involvement. On the other hand, choosing to diminish the degree of selfishness to prioritize family is essentially the same as choosing to sacrifice athletic performance. Jonathan explained, “I wouldn’t want to have a child when I was at the top of my game because it’s very selfish up there.” He further noted how becoming a parent amid an elite athletic career implies that “you do have to let go … of your … 100% ability in the sport.” Some of the participants also described this trade-off as a series of experiences, decisions, and an ebb and flow between these two extremes that were dependent on an array of factors such as the time of year in relation to competitive season, their child(ren)’s age(s), or future goals and/or previously accomplished career milestones (e.g., qualifying for an Olympic team), as evidenced below.

For many of the fathers, travelling on their own without their family was a way for them to prioritize their athletic commitments, especially around important competitions. Cameron, for example, preferred to travel on his own for major competitions:

When it came to major championships like World Championships and Olympics … I preferred for [my family] not to be there because it … would’ve just been one more thing for me to think about. … If they’re there, I’m gonna be thinking about them being in the right spot at the right time, making sure their tickets are fine, making sure they’re safe. And so it adds another level of stress … it’s another stressor that I’m not prepared to deal with.

Phil shared a very similar view:

There’s a risk … with, you know, what if [my child] does have an odd bad night and it’s two nights before [a competition]? I wouldn’t want to risk that. And you know, this is how I make a living … and I work really hard to get to this point. So, it just wouldn’t make sense to put that at risk.

A few of the fathers highlighted the advantages (to athletic performance) of traveling on their own not only for competitions but also for training camps, especially during the months leading up to a peak in their athletic seasons. Dennis shared that his daughter was six weeks old when he “packed up the bags and went to [training camp] for six months … to train and get ready for the Olympics.” He further elaborated on the importance of these types of decisions to mitigate the impediments to athletic performance that can be brought on by fatherhood:

I love my kids and I’m happy I had them during my career, but, at the same time, I was fully in race mode. So, when I was away on training trips or training camps, the last thing I’d want to do is bring a two-year-old screaming kid and take my focus off that … I didn’t want to take away from what I was doing [athletically].

However, Dennis also reflected on the difficulties that came with this decision: “That was a choice I made to have kids while I was competing, knowing that it was still going to be pretty hard for our family, with me being gone probably four to six months a year.”

Jake discussed what it was like leaving for his training camp nearly seven weeks following the birth of his daughter:

[My coach and I] made the decision to go away on [a training] camp … The first week was probably the hardest, probably the most emotional I’ve probably ever been ever, I think, being away from [my partner and child]. … I think looking back, I regret going away on [the training camp] only because of [my daughter’s] age. And it’s quite a lot on my partner to have to do everything else. However, it’s short-term sacrifice for long-term goal … that’s what I keep telling myself, anyway.

Jonathan touched on how today’s technology (e.g., FaceTime or video calls) can help to minimize the hardships of being away from family and loved ones; however, he stressed how these techniques are just “not the same, especially with that [young] age … [the child] can’t engage.” He provided a specific example of how “it was weird” when he realized that, upon his return home following the [anonymized] OlympicGames, his young son’s “voice had changed a little bit,” and he felt like he had to readjust to being around his own son. These fathers exemplified how their children’s ages can play an important part in the emotional toll they experienced and, in turn, represent a significant component in the trade-off between parenting and athletics.

Additionally, for some of the fathers, the emotional toll that they experienced came in the form of guilt, particularly because leaving for long periods of time meant that they were leaving the majority of parenting duties to fall on their partner/spouse. Colin said,

If I were to go away for any more than 10 days or two weeks, I think it would be too much of a burden … for my partner to be able to continue to do her job and to organize childcare for our children [because] our roles as parents are quite equal. And so … that additional burden on her … even if she said that she could take it on, I just wouldn’t feel comfortable. … I would just feel too guilty.

This same participant noted that it is simply not worth going through these forms of added stress to be able to fully prioritize athletic performance, in large part due to past accomplishments and the milestones that he had already accomplished throughout his running career: “I don’t think I’ll have many regrets based on having already been to the Olympics and stuff … if, you know, maybe I’m cutting a few corners and it doesn’t work out. I’m not that concerned.”

All the fathers in this study emphasized that both athletics and fatherhood not only represent two very significant areas in their lives, but that they are also two areas in which there are differing expectations to perform. These differing expectations contributed to these fathers’ experiences of having to negotiate their performances as elite athletes versus their performances as fathers.

## Discussion

Our findings make important contributions to literature on the experiences of elite athletes who combine their professional athletic careers with fatherhood. While the results support other scholars’ work on men’s experiences with fatherhood in a sport context (e.g., Coakley, 2006; Gottzen & Kremer-Sadlik, 2012; Graham & Dixon, 2014, 2017; McGannon et al., 2018; Pot & Keizer, 2016; Rynkiewicz et al., 2021), as well as on the experiences of elite athlete-parents (particularly mothers’; e.g., Darroch et al., 2016, 2019; Massey & Whitehead, 2022; McGannon et al., 2015; Palmer & Leberman, 2009), they also add important novelty and nuance.

### Elite Athletics, Fatherhood, and Caring Masculinities

The three themes that we constructed in this study produce elite male athletes as not only fathers, but as *involved* fathers – despite the first two themes contrasting one another (i.e., fatherhood improves vs. impedes elite athlete-fathers’ athletic performance). The fathers in this study exhibited differing approaches to balancing their fatherhood and athletic commitments, but none of the participants alluded to their parenting as a responsibility that they would ever disregard nor as something that is to be entirely attended to by their female partner/spouse. Rather, many of the athletes attested their commitments to being involved fathers and welcomed the new emotional experiences, from guilt to gratitude, that came with this pursuit.

In this study, the athletes’ constructions of themselves as involved fathers are in line with scholarly discussions around redefining masculinities through men’s more frequent involvement in fatherhood (Scheibling, 2020). Understanding fatherhood through a caring masculinities framework allows for the recognition of how most of the athletes in this study embraced values of care and, in doing so, engaged in a form of rejection of domination (Elliott, 2016). These understandings are also in line with new masculinities, which Lund et al. (2019) broadly referred to as the “increased involvement of men in caring practices and especially in fathering” (p. 1380). By examining our findings through a caring masculinities lens, our findings support men embracing new masculinities as a prevailing approach in disrupting gendered practices (Lund et al., 2019; Randles, 2018) by way of challenging ever-present narratives that support orthodox and hegemonic masculinity in sport (Adams et al., 2010; Stick, 2021).

By illustrating that men can indeed be both involved fathers and elite athletes, these sentiments contrast dominant societal narratives about “part-time fathers,” which position men as secondary parents to women (Stevens, 2015; Sunderland, 2000; Wall & Arnold, 2007), in addition to dominant societal family narratives that are rooted in the traditional gender role model that produces the work domain as being “more important for men than for women” (Cerrato & Cifre, 2018, p. 2), which insinuate that family is a burden to men’s work responsibilities. The participants in our study emphasized how fatherhood introduced them to a new sense of balance between having to let go of previous habits and routine while also gaining newfound motivation and “dad strength,” and how both these elements, in varying ways, influenced their athletic performance. This balance, in many respects, relates to the negotiation of responsibilities, sacrifices, and life changes in addition to the rewards and sense of fulfillment that many men feel when becoming new involved fathers (Deave & Johnson, 2008; Lewington et al., 2021; St John et al., 2005). Our findings demonstrate the capacity for men to be actively involved in family – and thus in roles that are beyond those directly related to being the “breadwinner” (Doucet, 2009, p. 89) – and how such involvement can foster positive life enrichment rather than conflict for men who are navigating both work and family roles.

### High Performance as an Athlete Versus High Performance as a Parent

A major finding in this study is that all the fathers emphasized a trade-off between their performances as an athlete and their roles as a father. Many of the fathers’ descriptions of this practice of give-and-take, where one side must be compromised for the other, were congruent with McGannon et al.’s (2018) results from their ethnographic content analysis of British professional tennis star Andy Murray’s journey as both a father and high-profile athlete. When the expectation of being a “good father” is constructed as a threat to athletic identity, elite athlete-fathers may experience what McGannon and colleagues referred to as a “push-pull” (p. 677) between work and family, which is often steered by differing expectations of masculinity in both areas (Doucet, 2004; Finn & Henwood, 2009). Such differing expectations are entrenched within traditional gendered beliefs that characterize men as both better suited to work and more likely to be involved in sport (de Haan & Knoppers, 2020), which construct elite male athletes as, first and foremost, full-time athletes and, secondly, part-time fathers (Stevens, 2015; Sunderland, 2000). The pressures and expectations brought on by such beliefs can limit the ways in which elite athlete-fathers manage the trade-off between fatherhood and athletics (Kangas et al., 2019).

Our findings place elite athlete-fathers in positions to be able to resist dominant narratives that reproduce the idea that fatherhood only impedes and does not improve athletic performance and, hence, that involved fatherhood is incompatible with high-performance sport (Cohen, 2016; McGannon et al., 2018). Many of the participants in our study described the trade-off between fatherhood and athletic performance as a dynamic process that is dependent on a multitude of factors that contribute to an ebb and flow of priority and time management between fathering and athletic commitments, including child(ren)’s age(s), future goals and/or previously accomplished career milestones, and time of year in relation to competitive season. In comparing these findings to those around female athletes who are pregnant/parenting, it is important to recognize how athlete-fathers may face similar – though gendered (and thus inherently different) – tensions between parent versus athlete identities (Darroch & Hillsburg, 2017; McGannon et al., 2015, 2018).

An extensive list of elements has been highlighted in the literature concerning the challenges associated with navigating motherhood as an elite athlete (Darroch et al., 2019; Darroch & Hillsburg, 2017; Davenport et al., 2022; Massey & Whitehead, 2022; McGannon et al., 2015; Palmer & Leberman, 2009; Tekavc et al., 2020). Included among this list is a wide range of barriers such as having to manage training loads throughout pregnancy and postpartum (Darroch et al., 2022a), potential postpartum injuries (Davenport et al., 2023), breastfeeding (if an athlete decides to do so; Giles et al., 2016), identity shifts between athlete versus mother (Darroch & Hillsburg, 2017; McGannon et al., 2015; Palmer & Leberman, 2009), lack of support from athletic governing bodies and/or sponsors (Darroch et al., 2019), and all sorts of differing media critiques – many of which either continue to reinforce hegemonic masculinity in sport or simply ignore female athletes altogether (Coche, 2017; McGannon et al., 2012, 2015; Scott et al., 2022; Toffoletti & Thorpe, 2018).

Our findings point to ways in which the contemporary era of involved fatherhood may come with a new reality wherein elite male athletes who are (involved) fathers find themselves wrestling with some, though not all, similar tensions to those faced by athlete-mothers. That being said, while we ought to recognize these parallels, it is simultaneously imperative that we also resist any urge to conflate the parenthood experiences of male and female athletes. Despite these seemingly analogous experiences, pregnant/parenting elite female athletes continue to be disadvantaged in sport (Scott et al., 2022), especially as the prevailing notion that motherhood is incompatible with elite sport is largely rooted in the clear biological differences between female and male athletes (Darroch et al., 2019). Although male athletes’ experiencing of a trade-off between athletic and fatherhood responsibilities is, in many ways, in line with female athletes’ own experiences of juggling mother- and athlete-related identities (Darroch & Hillsburg, 2017), it is nevertheless distinctively female athletes who must face the massive physical demands of both pregnancy and athletic performance in addition to uniquely facing potential psychological and emotional challenges including postpartum depression (Bø et al., 2017, 2018).

The bottom line is that pregnant and parenting athletes *all* require support to be able to perform at their best both as athletes and as parents – but the nature of such support must look different for female versus male athletes. For example, the potential introduction of policies around parental leave in elite athletics or access to childcare supports could be elements of change that could positively impact both female athletes who are pregnant and/or mothers as well as male athletes who are expectant and/or currently fathers. However, female athletes who are pregnant and/or mothers may also require additional provisions such as maternity protections in their contracts and access to proper medical assistance (e.g., prenatal care, obstetrician gynecologist, pelvic floor specialist, mental health practitioner during postpartum recovery; Smith et al., 2023; &Mother, 2021).

Our findings can open the door for key members across the athletics industry – including policymakers, sponsors, and athletics governing bodies – to take note of male elite athletes’ involvement in parenthood in addition to both the challenges as well as the benefits that children can bring to elite athlete-fathers’ careers in high-performance athletics. Recognition of these findings can lead to improved supports for pregnant/parenting elite athletes by way of reinforcing male athletes’ positionings as advocates for change within women’s sports (Lebel et al., 2021; Smith et al., 2023) and contributing to alleviating the burden of parenting responsibilities that is often still placed on athlete-mothers.

## Conclusions

There are a few important elements regarding this study that we feel must be acknowledged. First, all interviews that were conducted for this research took place amid the COVID-19 pandemic and thus took place virtually due to restrictions on gatherings. The COVID-19 pandemic marked a period of time not only when parents generally experienced varying levels of perceived stress (Adams et al., 2021; Washif et al., 2021), but also a time when pregnant/parenting elite athletes faced particularly unique stressors as they balanced the delayed Tokyo 2020 Olympic Games, disrupted training regimes, and parenting duties (Darroch et al., 2022b).

Additionally, given that the athletes who participated in this study are elite distance runners from three high-income countries and all had a partner/spouse, are abled-bodied, and all but one are white, we must acknowledge that our results lack geographical and cultural diversity. Further research that explores the different challenges and experiences of athletes who may not have access to the same resources or support, who may be subjected to different discursive practices (e.g., fathers who are single, racialized, or members of the LGBTQIA2 + community; Carroll, 2018), and/or who may compete within a different sport context is recommended. Furthermore, given that our findings in this manuscript were centred around the experiences of male athletes and the impact of children on their athletic careers, another avenue for future research could be the inverse of this relationship to explore understandings around how a career in high-performance athletics may impact male athletes’ roles as fathers.

In line with our methodological approach, future directions that stem from this work should also focus on the dissemination of research findings through the use of language that is both widely understandable as well as accessible (Israel et al., 1998). With that said, our research team is actively working with members of our CAB to co-author an article for a mainstream publication to highlight the findings of this work. Furthermore, we will also work to develop a policy brief to be presented to athletic governing bodies to underscore the importance of incorporating athlete-fathers in policy provisions for parental leave.

To our knowledge, this is the first study to consider the experiences of multiple fathers who are elite/international and world-class runners, in addition to being the first study in this area of research to apply the tenets of CBPR. Overall, our findings presented in this paper contribute to the literature on parenthood and elite sport and underscore the need to recognize the involvement of male elite athletes in parenthood as well as both the challenges and benefits that children can bring to athlete-fathers’ careers in elite sport. While there have been significant calls for a more profound emphasis to be placed on gender within discussions around parenthood and elite sport, the majority of scholarly attention has been directed towards female athletes, pregnancy, and maternity. In the name of gender equity, it is important that athlete-fathers are included within these conversations, too.

Through this study, we have highlighted how fatherhood may improve while also impede elite athlete-fathers’ athletic performance and, as a result, that these individuals face a trade-off in their experiences of navigating both athletics and fatherhood. Our findings thus underscore the ways in which fatherhood can impact male athletes and their high-performance athletic careers, how these experiences reflect the new era of involved fatherhood, and, importantly, how the trade-off between athletic performance and fatherhood responsibilities is analogous to some of the underlying identity tensions that have been thoroughly explored and reported with regard to the experiences of elite female athletes who are pregnant and/or mothers (Darroch & Hillsburg, 2017; McGannon et al., 2015, 2018; Palmer & Leberman, 2009). Recognizing male athletes’ involvement in parenthood and the parallels between fatherhood, motherhood, and elite sport may not only lead to better support for athlete-fathers but can also work towards diminishing the expectation that women are primarily involved in childcare responsibilities. This can, in turn, alleviate the significant challenges with which elite athlete-mothers have been frequently burdened through discriminatory policies and practices within the sports industry (Scott et al., 2022).

## Notes

1. According to McKay et al.’s (2022) athlete-participant classification framework, Elite/International includes athletes who have competed at the international level, top 4–300 in world rankings, and/or have achieved within 7% of world-record performance; World Class includes athletes who are/were Olympic and/or World Championship medalists, world-record holders, within 2% of world-record performance, top 3–20 in world rankings, and/or top 3–10 at an Olympics or World Championships.

## References

[bibr1-10608265231204564] Adams A. Anderson E. McCormack M. (2010). Establishing and challenging masculinity: The influence of gendered discourses in organized sport. Journal of Language and Social Psychology, 29(3), 278–300. 10.1177/0261927X10368833

[bibr2-10608265231204564] Adams E. L. Smith D. Caccavale L. J. Bean M. K. (2021). Parents are stressed! Patterns of parent stress across COVID-19. Frontiers in Psychiatry, 12, 626456. 10.3389/fpsyt.2021.626456 33897489 PMC8060456

[bibr3-10608265231204564] Andreasson J. Johansson T. Danielsson T. (2018). Becoming an Ironman triathlete. Extreme exercise, gender equality and the family puzzle. Sport in Society, 21(9), 1351–1363. 10.1080/17430437.2017.1388787

[bibr4-10608265231204564] Astone N. M. Peters H. E. (2014). Longitudinal influences on men’s lives: Research from the transition to fatherhood project and beyond. Fathering, 12(2), 161–173. 10.3149/fth.1202.161

[bibr5-10608265231204564] Baldwin S. Malone M. Sandall J. Bick D. (2018). Mental health and wellbeing during the transition to fatherhood: A systematic review of first-time fathers’ experiences. JBI Database of Systematic Reviews and Implementation Reports, 16(11), 2118–2191. 10.11124/JBISRIR-2017-003773 30289768 PMC6259734

[bibr6-10608265231204564] Bataille C. D. Hyland E. (2023). Involved fathering: How new dads are redefining fatherhood. Personnel Review, 52(4), 1010–1032. 10.1108/PR-06-2019-0295

[bibr7-10608265231204564] Blee K. M. Taylor V. (2002). Semi-structured interviewing in social movement research. In Klandermans B. Staggenborg S. (Eds.), Methods of social movement research (pp. 92–117). University of Minnesota Press.

[bibr8-10608265231204564] Bø K. Artal R. Barakat R. Brown W. J. Davies G. A. L. Dooley M. Evenson K. R. Haakstad L. A. H. Kayser B. Kinnunen T. I. Larsén K. Mottola M. F. Nygaard I. van Poppel M. Stuge B. Khan K. M. (2017). Exercise and pregnancy in recreational and elite athletes: 2016/17 evidence summary from the IOC expert group meeting, Lausanne. Part 3 – exercise in the postpartum period. British Journal of Sports Medicine, 51(21), 1516–1525. 10.1136/bjsports-2017-097964 28642221

[bibr9-10608265231204564] Bø K. Artal R. Barakat R. Brown W. J. Davies G. A. L. Dooley M. Evenson K. R. Haakstad L. A. H. Kayser B. Kinnunen T. I. Larsen K. Mottola M. F. Nygaard I. van Poppel M. Stuge B. Khan K. M. (2018). Exercise and pregnancy in recreational and elite athletes: 2016/2017 evidence summary from the IOC expert group meeting, Lausanne. Part 5 – Recommendations for health professionals and active women. British Journal of Sports Medicine, 52(17), 1080–1085. 10.1136/bjsports-2018-099351 29895607

[bibr10-10608265231204564] Boniface A. (2017, December 6). Special report: Athletes’ juggle can be a struggle. Athletics Weekly. https://athleticsweekly.com/performance/special-report-athletes-juggle-struggle-70575/

[bibr11-10608265231204564] Bowling A. (2014). Research methods in health: Investigating health and health services. McGraw-Hill Education.

[bibr12-10608265231204564] Braun V. Clarke V. (2006). Using thematic analysis in psychology. Qualitative Research in Psychology, 3(2), 77–101. 10.1191/1478088706qp063oa

[bibr13-10608265231204564] Braun V. Clarke V. (2019). Reflecting on reflexive thematic analysis. Qualitative Research in Sport, Exercise and Health, 11(4), 589–597. 10.1080/2159676X.2019.1628806

[bibr15-10608265231204564] Buchler S. Perales F. Baxter J. (2017). Does parenthood change attitudes to fathering? Evidence from Australia and Britain. Sex Roles, 77(9–10), 663–675. 10.1007/s11199-017-0757-8

[bibr16-10608265231204564] Carroll M. (2018). Gay fathers on the margins: Race, class, marital status, and pathway to parenthood: Gay fathers on the margins. Family Relations, 67(1), 104–117. 10.1111/fare.12300

[bibr17-10608265231204564] Cerrato J. Cifre E. (2018). Gender inequality in household chores and work-family conflict. Frontiers in Psychology, 9, 1330–1330. 10.3389/fpsyg.2018.01330 30123153 PMC6086200

[bibr18-10608265231204564] Chin R. Hall P. Daiches A. (2011). Fathers’ experiences of their transition to fatherhood: A metasynthesis. Journal of Reproductive and Infant Psychology, 29(1), 4–18. 10.1080/02646838.2010.513044

[bibr19-10608265231204564] Clarke V. Braun V. (2017). Thematic analysis. The Journal of Positive Psychology, 12(3), 297–298. 10.1080/17439760.2016.1262613

[bibr20-10608265231204564] Coakley J. (2006). The good father: Parental expectations and youth sports. Leisure Studies, 25(2), 153–163. 10.1080/02614360500467735

[bibr101-10608265231204564] Coche R. (2017). How athletes frame themselves on social media: An analysis of Twitter profiles. Journal of Sports Media, 12(1), 89–112. 10.1353/jsm.2017.0004

[bibr21-10608265231204564] Cohen D. T. (2016). Iron dads: Managing family, work and endurance sport identities. Rutgers University Press.

[bibr22-10608265231204564] Condon J. Boyce P. Corkindale C. J. (2004). The first-time fathers study: A prospective study of the mental health and wellbeing of men during the transition to parenthood. Australian and New Zealand Journal of Psychiatry, 38(1), 56–64. 10.1111/j.1440-1614.2004.01298.x 14731195

[bibr99-10608265231204564] Connell R. (1987). Gender and power: Society, the person, and sexual politics. Stanford University Press.

[bibr23-10608265231204564] Connell R. (1995). Masculinities. Polity Press.

[bibr24-10608265231204564] Connell R. Messerschmidt J. W. (2005). Hegemonic masculinity: Rethinking the concept. Gender & Society, 19(6), 829–859. 10.1177/0891243205278639

[bibr26-10608265231204564] Da Costa D. Danieli C. Abrahamowicz M. Dasgupta K. Sewitch M. Lowensteyn I. Zelkowitz P. (2019). A prospective study of postnatal depressive symptoms and associated risk factors in first-time fathers. Journal of Affective Disorders, 249(3), 371–377. 10.1016/j.jad.2019.02.033 30818245

[bibr27-10608265231204564] Darroch F. Giles A. Hillsburg H. McGettigan-Dumas R. (2019). Running from responsibility: Athletic governing bodies, corporate sponsors, and the failure to support pregnant and postpartum elite female distance runners. Sport in Society, 22(12), 2141–2160. 10.1080/17430437.2019.1567495

[bibr28-10608265231204564] Darroch F. Giles A. McGettigan-Dumas R. (2016). Elite female distance runners and advice during pregnancy: Sources, content, and trust. Women in Sport & Physical Activity Journal, 24(2), 170–176. 10.1123/wspaj.2015-0040

[bibr29-10608265231204564] Darroch F. Hillsburg H. (2017). Keeping pace: Mother versus athlete identity among elite long distance runners. Women’s Studies International Forum, 62(1), 61–68. 10.1016/j.wsif.2017.03.005

[bibr30-10608265231204564] Darroch F. E. Schneeberg A. Brodie R. Ferraro Z. Wykes D. Hira S. Giles A. Adamo K. Stellingwerff T. (2022a). Effect of pregnancy in 42 elite to world-class runners on training and performance outcomes. Medicine & Science in Sports & Exercise, 55(1), 93–100. https://pubmed.ncbi.nlm.nih.gov/35975937/35975937 10.1249/MSS.0000000000003025

[bibr31-10608265231204564] Darroch F. E. Smith S. V. M. Sheppard-Perkins M. Giles A. R. Wykes D. (2022b). Exploring the stress of Olympic postponement due to COVID-19 on elite/international and world class parenting and pregnant track athletes. Department of Health Sciences, Carleton University. [Manuscript submitted for publication]. 10.3389/fspor.2023.1001127 PMC10127675 37113985

[bibr32-10608265231204564] Darwin Z. Galdas P. Hinchliff S. Littlewood E. McMillan D. McGowan L. Gilbody S. (2017). Fathers’ views and experiences of their own mental health during pregnancy and the first postnatal year: A qualitative interview study of men participating in the UK born and bred in Yorkshire (BaBY) cohort. BMC Pregnancy and Childbirth, 17(1), 45–45. 10.1186/s12884-017-1229-4 28125983 PMC5270346

[bibr33-10608265231204564] Davenport M. H. Nesdoly A. Ray L. Thornton J. S. Khurana R. McHugh T.-L. F. (2022). Pushing for change: A qualitative study of the experiences of elite athletes during pregnancy. British Journal of Sports Medicine, 56(8), 452–457. 10.1136/bjsports-2021-104755 35135828 PMC8995814

[bibr34-10608265231204564] Deave T. Johnson D. (2008). The transition to parenthood: What does it mean for fathers? Journal of Advanced Nursing, 63(6), 626–633. 10.1111/j.1365-2648.2008.04748.x 18808584

[bibr35-10608265231204564] DeCarlo M. (2018). Scientific inquiry in social work. Pressbooks. https://scientificinquiryinsocialwork.pressbooks.com/chapter/10-2-sampling-in-qualitative-research/

[bibr100-10608265231204564] Davenport M. H. Ray L. Nesdoly A. Thornton J. Khurana R. McHugh T.-L. F. (2023). We’re not superhuman, we’re human: A qualitative description of elite athletes’ experiences of return to sport after childbirth. Sports Medicine, 53(1), 269–279. 10.1007/s40279-022-01730-y 35900698 PMC9331002

[bibr36-10608265231204564] de Haan D. Knoppers A. (2020). Gendered discourses in coaching high-performance sport. International Review for the Sociology of Sport, 55(6), 631–646. 10.1177/1012690219829692

[bibr37-10608265231204564] Dionigi R. A. Fraser-Thomas J. Logan J. (2012). The nature of family influences on sport participation in Masters athletes. Annals of Leisure Research, 15(4), 366–388. 10.1080/11745398.2012.744274

[bibr38-10608265231204564] Doucet A. (2004). “It’s almost like I have a job, but I don’t get paid”: Fathers at home reconfiguring work, care, and masculinity. Fathering, 2(3), 277–303. 10.3149/fth.0203.277

[bibr39-10608265231204564] Doucet A. (2009). Dad and baby in the first year: Gendered responsibilities and embodiment. The Annals of the American Academy of Political and Social Science, 624(1), 78–98. 10.1177/0002716209334069

[bibr40-10608265231204564] Elliott K. (2016). Caring masculinities: Theorizing an emerging concept. Men and Masculinities, 19(3), 240–259. 10.1177/1097184X15576203

[bibr41-10608265231204564] Elliott S. Powell R. Brenton J. (2015). Being a good mom: Low-income, black single mothers negotiate intensive mothering. Journal of Family Issues, 36(3), 351–370. 10.1177/0192513X13490279

[bibr42-10608265231204564] Felix A. (2019, May 22). Allyson Felix: My own nike pregnancy story. The New York Times. https://www.nytimes.com/2019/05/22/opinion/allyson-felix-pregnancy-nike.html

[bibr43-10608265231204564] Finn M. Henwood K. (2009). Exploring masculinities within men’s identificatory imaginings of first-time fatherhood. British Journal of Social Psychology, 48(3), 547–562. 10.1348/014466608X386099 19091163

[bibr44-10608265231204564] Florczak K. L. (2004). An exploration of the concept of sacrifice. Nursing Science Quarterly, 17(3), 195–200. 10.1177/0894318404266423 15200718

[bibr104-10608265231204564] Giles A. R. Phillipps B. Darroch F. E. McGettigan-Dumas R. (2016). Elite distance runners and breastfeeding: A qualitative study. Journal of Human Lactation, 32(4), 627–632. 10.1177/0890334416661507 27512011

[bibr45-10608265231204564] Gonçalves G. Sousa C. Santos J. Silva T. Korabik K. (2017). Portuguese mothers and fathers share similar levels of work-family guilt according to a newly validated measure. Sex Roles, 78(3–4), 194–207. 10.1007/s11199-017-0782-7

[bibr47-10608265231204564] Gottzen L. Kremer-Sadlik T. (2012). Fatherhood and youth sports: A balancing act between care and expectations. Gender & Society, 26(4), 639–664. 10.1177/0891243212446370

[bibr48-10608265231204564] Graham J. A. Dixon M. A. (2014). Coaching fathers in conflict: A review of the tensions surrounding the work-family interface. Journal of Sport Management, 28(4), 447–456. 10.1123/jsm.2013-0241

[bibr49-10608265231204564] Graham J. A. Dixon M. A. (2017). Work-family balance among coach-fathers: A qualitative examination of enrichment, conflict, and role management strategies. Journal of Sport Management, 31(3), 288–305. 10.1123/jsm.2016-0117

[bibr50-10608265231204564] Israel B. A. Schulz A. J. Parker E. A. Becker A. B. (1998). Review of community-based research: Assessing partnership approaches to improve public health. Annual Review of Public Health, 19(1), 173–202. 10.1146/annurev.publhealth.19.1.173 9611617

[bibr51-10608265231204564] Jackson T. Bostock E. L. Hassan A. Greeves J. P. Sale C. Elliott-Sale K. J. (2022). The legacy of pregnancy: Elite athletes and women in arduous occupations. Exercise and Sport Sciences Reviews, 50(1), 14–24. 10.1249/JES.0000000000000274 34669626

[bibr52-10608265231204564] Kangas E. Lämsä A. Jyrkinen M. (2019). Is fatherhood allowed? Media discourses of fatherhood in organizational life. Gender, Work and Organization, 26(10), 1433–1450. 10.1111/gwao.12352

[bibr54-10608265231204564] Keizer R. Dykstra P. A. Poortman A.-R. (2010). Life outcomes of childless men and fathers. European Sociological Review, 26(1), 1–15. 10.1093/esr/jcn080

[bibr55-10608265231204564] Knoester C. Eggebeen D. J. (2006). The effects of the transition to parenthood and subsequent children on men’s well-being and social participation. Journal of Family Issues, 27(11), 1532–1560. 10.1177/0192513X06290802

[bibr56-10608265231204564] Knoester C. Petts R. J. Eggebeen D. J. (2007). Commitments to fathering and the well-being and social participation of new, disadvantaged fathers. Journal of Marriage and Family, 69(4), 991–1004. 10.1111/j.1741-3737.2007.00426.x

[bibr57-10608265231204564] Kotila L. E. Kamp Dush C. M. (2013). Involvement with children and low-income fathers’ psychological well-Being. Fathering, 11(3), 306–326. 10.3149/fth.1103.306 25614731 PMC4299464

[bibr58-10608265231204564] Lebel K. Mumcu C. Pegoraro A. LaVoi N. M. Lough N. Antunovic D. (2021). Rethinking women’s sport research: Looking in the mirror and reflecting forward. Frontiers in Sports and Active Living, 3, 746441. 10.3389/fspor.2021.746441 34708200 PMC8542874

[bibr59-10608265231204564] Lee J. Y. Lee S. J. (2018). Caring is masculine: Stay-at-home fathers and masculine identity. Psychology of Men and Masculinity, 19(1), 47–58. 10.1037/men0000079

[bibr60-10608265231204564] Lewington L. Lee J. Sebar B. (2021). “I’m not just a babysitter”: Masculinity and men’s experiences of first-time fatherhood. Men and Masculinities, 24(4), 571–589. 10.1177/1097184X21993884

[bibr61-10608265231204564] Locke A. Yarwood G. (2016). Exploring the depths of gender, parenting and “work”: Critical discursive psychology and the “missing voices” of involved fatherhood. Community, Work & Family, 20(1), 4–18. 10.1080/13668803.2016.1252722

[bibr62-10608265231204564] Lund R. Meriläinen S. Tienari J. (2019). New masculinities in universities? Discourses, ambivalence and potential change. Gender, Work and Organization, 26(10), 1376–1397. 10.1111/gwao.12383

[bibr63-10608265231204564] Machin A. (2015). Mind the gap: The expectation and reality of involved fatherhood. Fathering, 13(1), 36–59. 10.3149/fth.1301.36

[bibr64-10608265231204564] Massey K. L. Whitehead A. E. (2022). Pregnancy and motherhood in elite sport: The longitudinal experiences of two elite athletes. Psychology of Sport and Exercise, 60(4), 102139. 10.1016/j.psychsport.2022.102139

[bibr102-10608265231204564] McGannon K. Curtin K. Schinke R. Schweinbenz A. (2012). (De)Constructing Paula Radcliffe: Exploring media representations of elite running, pregnancy and motherhood through cultural sport psychology. Psychology of Sport and Exercise, 13(6), 820–829. 10.1016/j.psychsport.2012.06.005

[bibr65-10608265231204564] McGannon K. Gonsalves C. Schinke R. Busanich R. (2015). Negotiating motherhood and athletic identity: A qualitative analysis of Olympic athlete mother representations in media narratives. Psychology of Sport and Exercise, 20, 51–59. 10.1016/j.psychsport.2015.04.010

[bibr66-10608265231204564] McGannon K. McMahon J. Price J. (2018). Becoming an athlete father: A media analysis of first-time father tennis star Andy Murray and the implications for identity. International Journal of Sport and Exercise Psychology, 16(6), 670–687. 10.1080/1612197X.2017.1313296

[bibr67-10608265231204564] McKay A. K. Stellingwerff T. Smith E. S. Martin D. T. Mujika I. Goosey-Tolfrey V. L. Sheppard J. Burke L. M. (2022). Defining training and performance caliber: A participant classification framework. International Journal of Sports Physiology and Performance, 17(2), 317–331. 10.1123/ijspp.2021-0451 34965513

[bibr68-10608265231204564] Meleagrou-Hitchens L. A. Willig C. (2022). Men’s experience of their transition to first-time fatherhood during their partner’s pregnancy: An interpretative phenomenological analysis. Journal of Men’s Health, 18(1), 15. 10.31083/jomh.2021.102

[bibr69-10608265231204564] Miller Y. D. Brown W. J. (2005). Determinants of active leisure for women with young children – An “ethic of care” prevails. Leisure Sciences, 27(5), 405–420. 10.1080/01490400500227308

[bibr70-10608265231204564] Montaño A. (2019, May 12). Nike told me to dream crazy, until I wanted a baby. The New York Times. https://www.nytimes.com/2019/05/12/opinion/nike-maternity-leave.html

[bibr71-10608265231204564] Moran J. Koslowski A. (2019). Making use of work-family balance entitlements: How to support fathers with combining employment and caregiving. Community, Work & Family, 22(1), 111–128. 10.1080/13668803.2018.1470966

[bibrmother_2021-10608265231204564] &Mother . (2021). Resource guide. &mother News.

[bibr72-10608265231204564] Muhammad M. Wallerstein N. Sussman A. L. Avila M. Belone L. Duran B. (2015). Reflections on researcher identity and power: The impact of positionality on community based participatory research (CBPR) processes and outcomes. Critical Sociology, 41(7–8), 1045–1063. 10.1177/0896920513516025 27429512 PMC4943756

[bibr73-10608265231204564] Oláh L. S. Vignoli D. Kotowska I. E. (2021). Gender roles and families. In Zimmermann K. F. (Ed.), Handbook of labor, human resources and population economics (pp. 1–28). Springer. 10.1007/978-3-319-57365-6_23-1

[bibr74-10608265231204564] Palmer F. Leberman S. (2009). Elite athletes as mothers: Managing multiple identities. Sport Management Review, 12(4), 241–254. 10.1016/j.smr.2009.03.001

[bibr75-10608265231204564] Park C. Kwon S. Y. (2019). What is a good father? The meaning of a good father through sports parenting in South Korea. Sport in Society, 22(8), 1346–1361. 10.1080/17430437.2019.1584185

[bibr76-10608265231204564] Patton M. Q. (1990). Qualitative evaluation and research methods (4th ed.). Sage.

[bibr77-10608265231204564] Philpott L. F. Savage E. FitzGerald S. Leahy-Warren P. (2019). Anxiety in fathers in the perinatal period: A systematic review. Midwifery, 76, 54–101. 10.1016/j.midw.2019.05.013 31176080

[bibr78-10608265231204564] Pot N. Keizer R. (2016). Physical activity and sport participation: A systematic review of the impact of fatherhood. Preventive Medicine Reports, 4, 121–127. 10.1016/j.pmedr.2016.05.018 27413672 PMC4929128

[bibr79-10608265231204564] Randles J. (2018). “Manning up” to be a good father: Hybrid fatherhood, masculinity, and U.S. responsible fatherhood policy. Gender & Society, 32(4), 516–539. 10.1177/0891243218770364

[bibr80-10608265231204564] Reid C. (2004). Advancing women’s social justice agendas: A feminist action research framework. International Journal of Qualitative Methods, 3(3), 1–15. 10.1177/160940690400300301

[bibr81-10608265231204564] Roberts L. W. (2013). Community-based participatory research for improved mental healthcare. A manual for clinicians and researchers. Springer. 10.1007/978-1-4614-5517-2

[bibr82-10608265231204564] Rynkiewicz K. M. Singe S. M. Eason C. M. (2021). Athletic trainers’ use of support systems for balancing roles as an athletic trainer and parent. Journal of Athletic Training, 57(3), 282–290. 10.4085/1062-6050-0681.20 PMC8935648 34038944

[bibr83-10608265231204564] Scambor E. Bergmann N. Wojnicka K. Belghiti-Mahut S. Hearn J. Gullvag Holter Ø. Gärtner M. Hrženjak M. Scambor C. White A. (2014). Men and gender equality: European insights. Men and Masculinities, 17(5), 552–577. 10.1177/1097184X14558239

[bibr84-10608265231204564] Scheibling C. (2020). “Real heroes care”: How dad bloggers are reconstructing fatherhood and masculinities. Men and Masculinities, 23(1), 3–19. 10.1177/1097184X18816506

[bibr85-10608265231204564] Scott T. Smith S. V. M. Darroch F. E. Giles A. R. (2022). Selling vs. supporting motherhood: How corporate sponsors frame the parenting experiences of elite and Olympic athletes. Communication & Sport, 1–22. 10.1177/21674795221103415 PMC10619167 37920688

[bibr86-10608265231204564] Smith S. V. M. Giles A. R. Darroch F. E. (2023). Pregnancy, parenthood, and elite athletics: “There’s a lot of work still yet to be done.” Sociology of Sport Journal, 1[ahead-of-print], 1–10. 10.1123/ssj.2022-0162

[bibr87-10608265231204564] Spowart L. (2021). Snowboarding, motherhood and mobility. Annals of Leisure Research, 24(2), 193–208. 10.1080/11745398.2019.1669472

[bibr88-10608265231204564] Stevens E. (2015). Understanding discursive barriers to involved fatherhood: The case of Australian stay-at-home fathers. Journal of Family Studies, 21(1), 22–37. 10.1080/13229400.2015.1020989

[bibr89-10608265231204564] Stick M. (2021). Conflicts in sporting masculinity: The beliefs and behaviors of Canadian male athletes. Journal of Men’s Studies, 29(3), 315–334. 10.1177/10608265211004579

[bibr90-10608265231204564] St John W. Cameron C. McVeigh C. (2005). Meeting the challenge of new fatherhood during the early weeks. Journal of Obstetric, Gynecologic, and Neonatal Nursing, 34(2), 180–189. 10.1177/0884217505274699 15781595

[bibr91-10608265231204564] Sunderland J. (2000). Baby entertainer, bumbling assistant and line manager: Discourses of fatherhood in parentcraft texts. Discourse & Society, 11(2), 249–274. 10.1177/0957926500011002006

[bibr92-10608265231204564] Taylor E. A. Huml M. R. Dixon M. A. (2019). Workaholism in sport: A mediated model of work-family conflict and burnout. Journal of Sport Management, 33(4), 249–260. 10.1123/JSM.2018-0248

[bibr93-10608265231204564] Tekavc J. Wylleman P. Cecić Erpič S. (2015). Perceptions of dual career development among elite level swimmers and basketball players. Psychology of Sport and Exercise, 21(1), 27–41. 10.1016/j.psychsport.2015.03.002

[bibr94-10608265231204564] Tekavc J. Wylleman P. Cecić Erpič S. (2020). Becoming a mother-athlete: Female athletes’ transition to motherhood in Slovenia. Sport in Society, 23(4), 734–750. 10.1080/17430437.2020.1720200

[bibr95-10608265231204564] Tichenor V. McQuillan J. Greil A. L. Contreras R. Shreffler K. M. (2011). The importance of fatherhood to U.S. married and cohabiting men. Fathering, 9(3), 232–251. 10.3149/fth.0903.232

[bibr103-10608265231204564] Toffoletti K. Thorpe H. (2018). Female athletes’ self-representation on social media: A feminist analysis of neoliberal marketing strategies in “economies of visibility.” Feminism & Psychology, 28(1), 11–31. 10.1177/0959353517726705

[bibr96-10608265231204564] Wall G. Arnold S. (2007). How involved is involved fathering? An exploration of the contemporary culture of fatherhood. Gender & Society, 21(4), 508–527. 10.1177/0891243207304973

[bibr97-10608265231204564] Washif J. A. Mohd Kassim S. F. A. Lew P. C. F. Chong C. S. M. James C. (2021). Athlete’s perceptions of a “quarantine” training camp during the COVID-19 lockdown. Frontiers in Sports and Active Living, 2(622858), 1–8. 10.3389/fspor.2020.622858 PMC7841328 33521634

[bibr98-10608265231204564] Wynter K. Francis L. M. Fletcher R. McBride N. Dowse E. Wilson N. Di Manno L. Teague S. Macdonald J. A. (2020). Sleep, mental health and wellbeing among fathers of infants up to one year postpartum: A scoping review. Midwifery, 88, 102738. 10.1016/j.midw.2020.102738 32521406

